# Emotion Differentiation in Current and Remitted Major Depressive Disorder

**DOI:** 10.3389/fpsyg.2021.685851

**Published:** 2021-09-01

**Authors:** Renee J. Thompson, Daphne Y. Liu, Ella Sudit, Matt Boden

**Affiliations:** ^1^Emotion and Mental Health Lab, Department of Psychological & Brain Sciences, Washington University in St. Louis, St. Louis, MO, United States; ^2^VA Palo Alto Health Care System, Palo Alto, CA, United States

**Keywords:** emotion differentiation, major depressive disorder, emotional granularity, remitted depression, experience sampling

## Abstract

People with current major depressive disorder (MDD) experience diminished emotion differentiation. We tested the hypothesis that this emotional disturbance is chronic and also characterizes those whose MDD has remitted. As our main aim, we examined emotion differentiation in conjunction with elevated negative and diminished positive emotional intensity, which are both cardinal symptoms of MDD. As an exploratory aim, we examined the predominant theoretical conceptualization that people low in emotion differentiation use more general state terms (e.g., bad) and fewer emotion terms (e.g., anger) to describe their emotional experience. Participants (assessed via diagnostic interview) included individuals who had current MDD (current depressed; *n* = 48), individuals whose MDD was in full remission (remitted depressed; *n* = 80), and healthy controls (*n* = 87). Participants also completed two self-report measures of depressive symptoms and reported momentary emotion repeatedly for 14 days via experience sampling, from which we computed emotion differentiation (i.e., intraclass correlation coefficient) and emotional intensity (i.e., average of the mean emotion ratings across surveys). Finally, participants described a momentary emotional experience via an open-response format, which was coded for the use of general state and emotion terms. Compared to the healthy control group, the current and remitted depressed groups showed similarly low levels of negative and positive emotion differentiation. These findings suggest that diminished emotion differentiation may be a stable characteristic of depressive disorders and a possible target for future prevention efforts. Diminished negative emotion differentiation was significantly associated with higher depressive symptoms as assessed by only one of the depression measures, though this finding did not hold after adjusting for negative emotional intensity. Finally, participants’ emotion differentiation was not associated with use of general state and emotion terms, and groups did not use general state and emotion terms in ways that were consistent with the predominant theoretical conceptualization of emotion differentiation, suggesting the need for clarification in this research domain.

## Introduction

Major depressive disorder (MDD) is among the most prevalent and debilitating mental disorders (Eaton et al., 2012), and the already high prevalence rate is increasing (Weinberger et al., 2018). It is a highly recurrent disorder (Bockting et al., 2015), with more people experiencing recurrent episodes than single episodes (e.g., Andrade et al., 2003). These prevalence rates highlight the need to improve prevention efforts, including identifying possible risk factors. Depressive episodes are characterized by various disturbances in emotion (e.g., Houben et al., 2015; Liu and Thompson, 2017). Examining these emotional disturbances that characterize depressive episodes that are in remission could help identify risk factors associated with the onset and recurrence of MDD, informing primary and secondary prevention efforts.

One emotional disturbance that may confer risk for MDD is low emotion differentiation (hereafter differentiation; Barrett et al., 2001; Demiralp et al., 2012). Individuals with low differentiation are theorized to use general state terms (e.g., good, bad) to describe their feeling states and not to discern nuances between distinct emotions, whereas individuals with high differentiation are theorized to use emotion terms to describe how they feel and to discern the nuances between distinct emotions (e.g., sad versus angry; e.g., Boden et al., 2013; Dixon-Gordon et al., 2014; Erbas et al., 2014; Kashdan et al., 2015). Researchers most commonly measure differentiation using repeated measurements of precise emotion terms and compute a statistic, such as an intraclass correlation coefficient (ICC) (Thompson et al., 2021b). In this case, individuals with low differentiation are theorized to use similar terms over time to describe their feeling states, whereas those with high differentiation report varying combinations of emotions over time to describe their feeling states.

Differentiation of negative emotions (NED) and positive emotions (PED) have often been examined separately. Higher differentiation, particularly NED, has been shown to be adaptive, and it is associated with greater psychological well-being and reduced engagement with maladaptive behaviors (Erbas et al., 2014; Seah and Coifman, 2021). Researchers have proposed that low differentiation could lead to increases in depressive psychopathology via difficulty with emotion regulation, which characterizes MDD (e.g., Ottenstein, 2020). For example, people with low differentiation may have difficulty utilizing the nuanced information provided by emotions to effectively engage in emotion regulation, such as selecting the appropriate emotion regulation strategies (Barrett et al., 2001; Kashdan et al., 2015). Difficulties with emotion regulation have been theorized to lead to increased depressive psychopathology (e.g., Gross and Muñoz, 1995; Ottenstein, 2020). Consistent with this, evidence has linked low differentiation with increases in depressive symptoms over time (Rieffe and De Rooij, 2012; Liu et al., 2020). This led us to theorize that low differentiation may play a role in the etiology of depressive psychopathology and may exist outside of active depressive episodes. Therefore, we posit that low differentiation is a chronic feature of MDD and expect that diminished differentiation will characterize those whose MDD is in remission.

Most existing research on differentiation and depression among adults has focused on NED. Most studies have found that higher NED was associated with lower depressive symptoms (Erbas et al., 2014, Studies 2 and 3; Plonsker et al., 2017; Starr et al., 2017; Liu et al., 2020). Grühn et al. (2013) and Matt et al. (2016) did not find a significant association between NED and depressive symptoms, with Matt et al. (2016) speculating that the null finding may be due to their sample having low levels and a restricted range of current depressive symptoms. Consistent with this speculation, lower NED was significantly associated with higher depressive symptoms in adults with MDD (Goldston et al., 1992), as well as samples with elevated depressive symptoms or a sizable portion reporting clinically significant depressive symptoms (Starr et al., 2017; Liu et al., 2020; Ottenstein, 2020). Further, adults with current MDD had lower NED compared to healthy controls (Demiralp et al., 2012). Taken together, it appears that lower NED was more consistently associated with higher depressive symptoms when the samples had elevated and/or a wide range of depressive symptomatology. However, a significant inverse association between NED and depressive symptoms has been found in relatively healthy samples (e.g., Willroth et al., 2020), suggesting that factors other than the range of depressive symptoms may explain the mixed results, such as the use of different depression measures. Given the heterogeneity of the samples and designs of existing research, it is challenging to detect any pattern related to depression measure type, however.

In contrast to NED, there has been less research on depression and PED in adult samples (O’Toole et al., 2019; Liu et al., 2020; Thompson et al., 2021b), although the findings have been more consistent. PED has not been significantly related to depressive symptoms (Grühn et al., 2013; Matt et al., 2016; Starr et al., 2017; Liu et al., 2020). Similarly, adults with current MDD did not differ from healthy controls in PED (Demiralp et al., 2012). However, given that only one study has examined PED in those with MDD, it is critical to examine whether findings replicate. The role of PED versus NED in psychopathology is much less clear, though there are reasons to believe that PED is indeed related to adaptive emotion responding (e.g., Fredrickson, 2001; Shiota et al., 2014). Thus, more research on PED and psychopathology, more generally, is needed (Thompson et al., 2021b).

The central aim of the study was to examine differentiation in current and remitted MDD. We assessed differentiation via experience sampling, a method with good ecological validity. Experience sampling also minimizes retrospective recall bias (Schwarz, 2012), which is critical in depressed samples who are characterized by several negative cognitive biases (e.g., Gotlib and Joormann, 2010). Although most research on differentiation and depression has focused on depressive symptoms among relatively healthy samples (e.g., Liu et al., 2020), we recruited participants representing a wide range of depressive psychopathology: current MDD, remitted MDD (i.e., experienced a depressive disorder in the past but not currently), and a healthy control group. Groups were identified via diagnostic interviewing, the gold standard method of assessing depressive disorders instead of only assessing depressive symptoms using self-report measures, which tend to have low specificity and assess constructs that are not unique to depression (e.g., general distress; Bredemeier et al., 2010). By recruiting individuals at different stages of MDD (in and outside of depressive episodes), we included participants who represent much of the spectrum of the disorder and vary in their levels of current depressive symptoms, which we assessed using two self-report measures. In addition, examining differentiation among those whose MDD is in remission will inform whether diminished NED is a chronic feature of MDD, which could provide more insight into the role of NED in the etiology of MDD.

We examined differentiation in conjunction with emotional intensity, as high negative and low positive emotional intensity are primary symptoms of MDD (American Psychiatric Association, 2013). Replicating existing work on negative emotional intensity in MDD (e.g., Watson et al., 1988) and NED (Demiralp et al., 2012), we expected that the current depressed group would have higher negative emotional intensity and lower NED than the healthy control group. In terms of remitted MDD, research has found that people whose MDD is in remission experience higher negative emotional intensity than healthy controls (e.g., Wichers et al., 2012), and lower negative emotional intensity than those with current depressive disorders (Schoevers et al., 2020). Because we expected that diminished NED is relatively chronic and not only a state effect of being in a depressive episode, we hypothesized that the current and remitted depressed group would both have diminished NED relative to the healthy control group. This investigation will be the first to examine NED in remitted MDD, which could help inform whether NED may be a risk factor for MDD.

Based on the diagnostic criteria of MDD (American Psychiatric Association, 2013) and research on positive emotional intensity and PED, we expected that the current depressed group would have significantly lower positive emotional intensity (e.g., Watson et al., 1988) but similar levels of PED (Demiralp et al., 2012) relative to the healthy control group. Most evidence suggests that those with remitted MDD do not differ from healthy controls in positive emotional intensity (e.g., Wichers et al., 2012). Furthermore, those with remitted MDD have been found to have higher positive emotional intensity than those with current MDD (Schoevers et al., 2020). Regarding PED, we did not expect that the remitted depressed group would differ from the healthy control group or current depressed group based on existing literature investigating depressive psychopathology and PED. Although we did not expect group differences in PED, this study nevertheless contributes to the literature by examining PED in remitted depression for the first time.

The present study is also novel because of its exploratory aim focused on testing one tenet of the predominant theoretical conceptualization of differentiation–the use of general state and emotion terms. As in the present study, most researchers have measured differentiation using experience sampling (Thompson et al., 2021b), where participants are repeatedly prompted to rate the extent to which they feel a given list of emotion terms for a number of days or weeks. Researchers often compute ICCs between emotion terms to index trait differentiation (Thompson et al., 2021b), with high intercorrelation indicating low differentiation. However, this method does not allow researchers to test an important tenet of the predominant differentiation theory–whether low differentiation is characterized by a greater likelihood of using general state terms and a lower likelihood of using emotion terms (Thompson et al., 2021b). To address this limitation, we administered an online survey that assessed participants’ momentary emotional experiences using a free-response format. Assessing how these open-ended responses are correlated with differentiation using ICCs derived from experience sampling data allowed us to explicitly examine whether differentiation corresponds with how it is predominantly conceptualized (i.e., use of general state and emotion terms). Additionally, we examined whether the current depressed group, who has been shown to have diminished NED relative to a healthy control group (Demiralp et al., 2012), would be more likely to use general state terms and less likely to use emotion terms when describing their momentary emotional experiences–a pattern that would be consistent with the predominant theoretical conceptualization of differentiation. If the remitted depressed group reflects the current depressed group in terms of diminished NED, we would also expect them to show the same pattern in their use of general state and emotion terms relative to the healthy control group.

Finally, one might reasonably argue that a higher verbal ability would be associated with higher differentiation. However, Ottenstein and Lischetzke (2020) found that NED (assessed using ICCs) was associated with verbal ability to a small and non-significant degree. Further, Ottenstein and Lischetzke (2020) did not find a significant association between verbal ability and their open-ended measure of NED (i.e., specificity index) either. It is important to see if this pattern of findings replicate and examine the associations between verbal ability and PED. Consequently, we administered the vocabulary subtest of the Wechsler Adult Intelligence Scale–Third Edition (WAIS-III; Weschler, 1997) as a proxy for verbal ability to examine its associations with differentiation and with the use of general state and emotion terms.

## Materials and Methods

### Participants

The sample included 215 participants recruited for a study on everyday emotions and decision making. Participants were recruited from participant registries, ads (e.g., Craigslist), and fliers posted at local businesses and clinics. The sample was composed of 66.0% women and 34.0% men, with an average age of 44.3 years (*SD* = 16.1). Racial/ethnic composition included 69.8% White, 19.5% Black, 2.8% Asian, 0.5% Native American, and 7.0% other or multiracial (0.5% did not report). In addition, 1.4% reported that they were Latinx/a/o. Participants were generally highly educated with the following levels of education: bachelor’s degree (32.6%), a graduate or professional degree (32.1%), some college (24.2%), some high school or high school diploma (9.8%), and unknown (1.4%). In terms of employment status, 19.1% were employed part-time, 40.9% were employed full-time, 14.4% were retired, and 10.7% were unemployed; others were on disability, stay-at-home parents, and so forth.

Eligibility criteria for the study included speaking English as a primary language and not having severe visual or hearing impairments. In addition, individuals needed to meet criteria for one of three groups as defined by the Diagnostic and Statistical Manual of Mental Disorders (5th ed.; DSM-5; American Psychiatric Association, 2013). For the current depressed group (*n* = 48), individuals needed to meet criteria for a current major depressive episode in the context of MDD or persistent depressive disorder (regardless of the number of previous major depressive episodes). For the remitted depressed group (*n* = 80), individuals needed to meet criteria for at least two fully remitted depressive episodes in the context of MDD or persistent depressive disorder. For the healthy control group (*n* = 87), individuals were required to have no current or past mood or anxiety disorders. Inter-rater reliability scores showed that raters demonstrated perfect agreement in assessing the presence of current MDD, current persistent depressive disorder, past MDD, and past persistent depressive disorder (*k* = 1.0 for each) in a random subset of interviews (*n* = 48). Exclusionary criteria included current or past diagnoses of bipolar I, bipolar II, cyclothymic disorder, and current or past psychotic symptoms. Due to the high rate of comorbidity between depressive and anxiety disorders (Kessler et al., 2003), individuals with comorbid anxiety disorders were eligible for the two depressed groups, resulting in 70.8% of the participants in the current depressed group and 18.8% in the remitted depressed group meeting criteria for at least one comorbid anxiety disorder.

### Procedures

Interested individuals completed an initial telephone screen conducted by a post-baccalaureate project manager or an undergraduate research assistant, who briefly assessed participants’ experiences with the two cardinal symptoms of MDD (i.e., depressed mood and anhedonia, American Psychiatric Association, 2013). Individuals who were deemed as likely to be eligible for the study completed a series of self-report measures administered online (i.e., home survey) before attending a laboratory session, during which their eligibility would be more thoroughly assessed. In the laboratory session, participants completed Modules B/C (Psychotic Screening), D (Mood Disorders), and F (Anxiety Disorders) of the Structured Clinical Interview for DSM-5.0 (SCID-5-RV; First et al., 2015). Interviews were conducted by clinical psychology graduate students who had completed a graduate-level assessment course in which they learned to administer the SCID-5-RV. Interviewers obtained telephone supervision from the first author, a licensed psychologist, as needed. Participants completed two self-report measures of depressive symptoms–the anhedonic depression scale of the Mood and Anxiety Symptom Questionnaire (MASQ) (Watson et al., 1995) and the Center for Epidemiologic Studies Depression Scale (CES-D) (Radloff, 1977) before the SCID-5-RV interview.

Participants who met criteria for any of the three groups also completed additional self-report measures, cognitive tasks, including the WAIS-III vocabulary subtest (Weschler, 1997), and a 30-min individual experience sampling tutorial during the laboratory session. For the tutorial, undergraduate experimenters helped participants install the experience sampling software on their own iPhones or provided participants with a 4th-generation iPod Touch. We used the Status/Post iOS app developed by Christopher Metts, M.D., which collects data offline, obviating the need for Wi-Fi or a smartphone. The tutorial also included a presentation with slides and a full practice survey. Throughout the tutorial, experimenters assessed whether the participant understood the procedure and provided standardized examples. At the end of the session, participants were financially compensated for the home survey ($6) and for the laboratory session ($12/h). Participants who attended the laboratory session via public transportation received additional compensation for associated costs ($4 if traveled by bus or $5 if traveled by rail).

During the 14-day sampling period, which started the day after the laboratory session, participants were randomly prompted to complete five surveys a day for a total of 70 surveys. Participants chose the 15-h window during which they would complete surveys, and prompts occurred at random times within five 3-h windows per day. Participants had up to 15 min to start the survey before the survey closed, in which case data were marked as missing. Surveys occurred at an average of 3 h, 0 min, and 18 s apart (*SD* = 1 h, 1 min, 35 s). The mean percentage of surveys completed was 74.8% (*SD* = 18.3%, range = 20.0–99.0%). Groups did not differ in the time between surveys, *F*(2) = 0.20, *p* = 0.82, or the percentage of surveys completed, *F*(2) = 0.30, *p* = 0.74. The sample of 215 did not include 22 participants who experienced app problems (*n* = 7), withdrew (*n* = 7), had completed less than 20.0% of the surveys (*n* = 7), or whose behavior evoked concern about the validity of the data (*n* = 1). To encourage compliance, participants were called a few days into the sampling period to trouble-shoot problems. After the sampling period, participants were debriefed via email and financially compensated for the experience sampling portion ($40), with an additional bonus of $10 for completing at least 80.0% of surveys.

Target sample sizes were pre-determined to ensure sufficient power to examine hypotheses using multilevel modeling analyses for other study hypotheses. For the current study, *post hoc* power analyses of our central hypotheses tested using a multivariate analysis of variance (MANOVA). Follow-up analyses of variance (ANOVA) revealed adequate power given observed sample and effect sizes (Range = 0.78–0.99; Faul et al., 2007).

### Measures

#### Emotional Intensity

At each experience sampling survey, participants rated their momentary levels of emotion. Using a five-point scale ranging from 0 (*not at all*) to 4 (*extremely*), participants indicated the extent to which they were currently feeling a series of emotions using the following format: “I felt [EMOTION] at the time of the beep.” Emotions included low, moderate, and high arousal emotions from the affective circumplex (Barrett and Russell, 1999). Mean levels of negative emotion (i.e., bored, sluggish, sad, frustrated, nervous, and angry) and positive emotion (i.e., relaxed, content, calm, happy, excited, and enthusiastic) were computed for each survey, and these scores were averaged, creating an overall mean for negative emotion and for positive emotion. Importantly, research has shown that aggregating state measurements of a construct (e.g., emotional intensity) is often superior to assessing global measures of the same construct (Augustine and Larsen, 2012). As recommended by Nezlek (2017), we computed the mixed modeling functional equivalent to Cronbach’s α for negative and positive emotional intensity, which were 0.65 and 0.74, respectively. The ICC for negative emotional intensity was 0.42, meaning that 42 and 58% of the variance was at the between- and within-person-levels, respectively. The ICC for positive emotional intensity was 0.43.

#### Emotion Differentiation

We assessed differentiation using experience sampling data and computed differentiation following past research (e.g., Erbas et al., 2014). First, we computed the average ICC measuring consistency (Shrout and Fleiss, 1979) between negative emotions (i.e., bored, sluggish, sad, frustrated, nervous, and angry) and between positive emotions (i.e., relaxed, content, calm, happy, excited, and enthusiastic) across the experience sampling surveys, resulting in NED and PED, respectively. Based on 2 considerations, 14 participants (11 healthy controls, 3 current depressed) with negative ICC values obtained from ratings of negative emotions were re-coded as having a value of zero rather than excluding them from analyses. First, theorists have stated that negative ICC values can be interpreted as representing low agreement between ratings (Giraudeau, 1996; Taylor, 2010). Second, in our data set, participants with negative ICC values and with lower ICC values (0–0.50) were similar in (a) the average number and percent of zero responses per prompt, and (b) average levels of negative affect intensity and variance per prompt. Negative ICC values may have resulted in part from participants responding to fewer prompts (relative to participants with positive, high and low ICC values). Thus, like participants with low ICC values, we considered participants with negative ICC values as having high emotion differentiation. Then we transformed the ICC values using a Fisher’s r-to-z transformation (Pond et al., 2012). Finally, because higher ICC values reflect greater similarity in ratings of different emotions across occasions (i.e., lower differentiation), we subtracted the transformed scores from one, so that higher scores reflected greater differentiation to ease interpretation.

#### Depressive Symptoms

##### Mood and anxiety symptom questionnaire (MASQ)–anhedonic depression

Depressive symptoms were measured using the anhedonic depression subscale (22 items) of the MASQ (Watson et al., 1995). The MASQ anhedonic depression scale focuses on aspects of depressive psychopathology that uniquely characterize depression–anhedonia (e.g., “felt like nothing was enjoyable”) and low positive affect (e.g., “felt cheerful;” reverse-coded). It has been found to be psychometrically distinct from anxiety symptoms (Watson et al., 1995; Nitschke et al., 2001). Participants reported the extent to which they experienced depressive symptoms over the preceding week using a five-point Likert scale ranging from 1 (*not at all*) to 5 (*extremely*). A composite anhedonic depression scale score was computed for each participant by summing the 22 individual item scores, with the 14 items focusing on positive affect reverse-scored to reflect low positive affect. Higher scores indicate greater severity of depressive symptoms. The MASQ anhedonic depression scale has strong psychometric properties in community (Watson et al., 1995; Nitschke et al., 2001) and clinical samples (e.g., Watson et al., 1995). Internal reliability of the MASQ anhedonic depression subscale was excellent (α = 0.96).

##### Center for epidemiological studies depression scale (CES-D)

Depressive symptoms were also assessed using the 20-item CES-D (Radloff, 1977). The CES-D covers a wide range of depressive symptoms, including depressed affect (e.g., “I feel depressed”), lack of positive affect (e.g., “I feel hopeful about the future;” reverse-coded), somatic complaints (e.g., “I did not feel like eating; my appetite was poor”), and interpersonal concerns (e.g., “I felt that people dislike me”). Participants rated the frequency at which they had experienced each symptom over the preceding week using a four-point Likert scale ranging from 0 (*rarely or none of the time*) to 3 (*most or all of the time*). A composite CES-D score was computed for each participant by summing their scores of the 20 individual items, four of which were reverse-coded. Higher CES-D scores indicate greater severity, with scores equal or greater than 16 suggesting symptom severity of clinical significance (Radloff, 1977). The CES-D was developed to assess depressive symptoms of community samples, demonstrating adequate reliability and validity (Eaton et al., 2004), and has been validated in clinical samples (Weissman et al., 1977; Morin et al., 2011). Internal reliability of the CES-D was excellent (α = 0.94).

#### General State and Emotion Terms

As part of the home survey, participants completed an open-ended measure assessing momentary emotional experience. They were presented with the instructions, “Please answer with as much detail as you need to describe your feelings,” before being asked to “Describe how you feel right now” by typing their responses in a textbox. For each response, we identified terms describing general states (i.e., vague, general, diffuse, or generic feeling states) and emotions (multifaceted, embodied phenomena that involve loosely coupled changes in subjective experience, behavior, and peripheral physiology; Barrett et al., 2007). We coded these terms into eight categories: (1) positive general states (e.g., good, wonderful), (2) negative general states (e.g., bad, awful), (3) mixed general states (e.g., mixed, ambivalent), (4) neutral general states (e.g., fine, ok, so-so), (5) positive emotions (e.g., happy, excited), (6) negative emotions (e.g., sad, angry), (7) mixed emotions (e.g., bittersweet), and (8) neutral emotions (e.g., surprise). All eight categories were binary coded (e.g., someone who used one or more positive emotions would be coded as having a “one” for the positive emotion category). No participants used mixed general state or emotion terms, so these were dropped from further analyses. Two advanced undergraduate research assistants, both of whom were blind to participants’ group status, independently scored each response with disagreements in ratings resolved through discussion with the first author. Consensus ratings were used. Interrater reliability, as measured by percent agreement between raters (McHugh, 2012), was excellent for the eight term categories (Range = 97.0–100.0%).

#### Verbal Ability

We administered the WAIS-III vocabulary subtest (Weschler, 1997) as a proxy for verbal ability or verbal intelligence quotient (IQ) as in Muhtadie et al. (2015). The subtest was administered via MediaLab software on a desktop computer, with the experimenter reading the instructions that were visible to the participant. Participants were asked to define each word, which were presented one at a time. The experimenter left the room while the participant was given 4 min to define as many of the 26 words as possible. Then undergraduate research assistants scored each definition as a 0, 1, or 2, for a total composite score with a range of 0–52. After practicing on data from ten participants, the research assistants individually coded data from 85 participants. Then they met with a graduate student who had experience coding this task to arrive at consensus ratings for any disagreements; kappas (one per vocabulary word, total of 26 words) ranged from 0.81 to 1.00 (*M* = 0.92; *SD* = 0.05). After high reliability was established, the remaining data were coded by one of the undergraduate research assistants. Internal consistency of the items was also good (α = 0.81).

## Results

### Demographic Data by Group

First, we examined whether demographic and clinical characteristics differed by group, using ANOVA and chi-squared tests. There were no group differences in age, *F*(2, 212) = 0.72, *p* = 0.49, gender, χ^2^(2, *N* = 215) = 4.83, *p* = 0.09, distribution by race/ethnicity, χ^2^(8, *N* = 214) = 6.04, *p* = 0.64, or distribution by Latino/a/x, χ^2^(2, *N* = 215) = 1.43, *p* = 0.43. The three groups did not differ in the highest level of education completed, χ^2^(6, *N* = 212) = 7.96, *p* = 0.24, or employment status, χ^2^(16, *N* = 212) = 23.26, *p* = 0.11. The three groups significantly differed in levels of depressive symptoms as assessed by the MASQ anhedonic depression scale, *F*(2, 210) = 63.87, *p* < 0.001 (current depressed: *M* = 78.5, *SD* = 15.5; remitted depressed: *M* = 56.5, *SD* = 16.0; healthy control: *M* = 48.0, *SD* = 13.5), which is consistent with previous work (e.g., Figueroa et al., 2018). Importantly, the mean of the healthy control group was similar to levels reported in community samples (e.g., Bredemeier et al., 2010), and the means of the remitted depressed and healthy control groups were well below an established clinical cutoff of 76 (Buckby et al., 2007). We see a similar pattern of depressive symptoms by group for the CES-D measure too: The groups significantly differed in CES-D scores, *F*(2, 210) = 147.0, *p* < 0.001 (current depressed: *M* = 33.40, *SD* = 10.05; remitted depressed: *M* = 13.33, *SD* = 9.64; healthy control: *M* = 7.34, *SD* = 6.27). The groups also significantly differed in verbal ability, *F*(2, 208) = 3.69, *p* = 0.027, η*_*p*_*^2^ = 0.034; a *post hoc* Tukey test showed that the remitted depressed group (*M* = 27.90, *SD* = 8.84) scored significantly higher than the healthy control group (*M* = 24.96, *SD* = 9.73), *p* = 0.042, as well as than the current depressed group (*M* = 23.68, *SD* = 8.71), *p* = 0.013; the healthy control group and the current depressed group did not differ from each other in verbal ability, *p* = 0.444.

### Measure Descriptives and Correlations

Across the entire sample, NED ranged from −0.65 to 1.00 (*M* = 0.50, *SD* = 0.27), and PED ranged from −1.70 to 0.83 (*M* = −0.09, *SD* = 0.31). The low and negative values of differentiation scores were consistent with previous work (e.g., Dixon-Gordon et al., 2014; Lennarz et al., 2018; Widdershoven et al., 2019). Negative emotional intensity ranged from 0 to 2.04 (*M* = 0.47, *SD* = 0.37), and positive emotional intensity ranged from 0.06 to 3.01 (*M* = 1.51, *SD* = 0.62). Before testing our main hypotheses, we examined Spearman’s correlations between differentiation and emotional intensity (see Table 1). Negative emotional intensity and NED were significantly inversely associated, as were positive emotional intensity and PED. The small-to-moderate size of these correlations indicate that emotional intensity and differentiation have substantial unshared variance and are thus distinct constructs.

**TABLE 1:** Spearman’s correlations between emotion differentiation, emotional intensity, depressive symptoms, and verbal ability.

	NED	Negative emotional intensity	PED	Positive emotional intensity	Depressive symptoms: MASQ	Depressive symptoms: CES-D
NED	−					
Negative emotional intensity	−0.38**	−				
PED	0.17*	–0.04	−			
Positive emotional intensity	0.05	−0.22*	−0.15*	−		
Depressive symptoms: MASQ	–0.07	0.45**	0.09	−0.52**	−	
Depressive symptoms: CES-D	−0.23**	0.54**	0.07	−0.47**	0.83**	−
Verbal ability	0.06	0.16*	–0.10	–0.06	–0.05	–0.08

CES-D = the Center for Epidemiological Studies Depression Scale; MASQ = the anhedonic depression subscale of the Mood and Anxiety Symptom Questionnaire; NED = negative emotion differentiation; PED = positive emotion differentiation. *p < 0.05 (two-tailed), **p < 0.01 (two-tailed).

To test whether differentiation was associated with depressive symptoms in our sample, we computed the correlations of differentiation with the CES-D and with the MASQ anhedonic depression scale (see Table 1). Lower NED was significantly associated with higher depressive symptoms as measured by the CES-D, but not the MASQ anhedonic depression scale. PED showed a more consistent pattern in that it was not associated with either depressive symptom measure. Given that both NED and CES-D were significantly correlated with negative emotional intensity (Table 1) and based on existing concerns about the unique explanatory power of differentiation beyond emotional intensity (i.e., mean affect) in predicting well-being indices (Dejonckheere et al., 2019), we further examined the association between NED and CES-D scores controlling for negative emotional intensity. Results showed that the NED was no longer significantly associated with CES-D scores after accounting for negative emotional intensity, *b* = −3.19, *p* = 0.28.

Verbal ability was associated with NED and PED to a small and non-significant degree. Regarding the open-ended responses, verbal ability was significantly negatively correlated with positive general state terms, *r* = −0.15, *p* = 0.04, but it was uncorrelated with negative, *r* = −0.02, *p* = 0.81, neutral, *r* = 0.004, *p* = 0.95, or overall (across valence) general state terms, *r* = −0.09, *p* = 0.21. Additionally, verbal ability was significantly positively correlated with negative emotion terms, *r* = 0.16, *p* = 0.03, and overall (across valence) emotion terms, *r* = 0.19, *p* = 0.007, but it was uncorrelated with positive, *r* = 0.09, *p* = 0.20, or neutral emotion terms, *r* = 0.06, *p* = 0.39.

### Experience Sampling Data

#### Testing Group Differences in Differentiation and Emotional Intensity (Aim 1)

To assess group differences in differentiation and emotional intensity, we used a MANOVA. In terms of Pillai’s trace, there was a significant effect of group on NED, negative emotional intensity, PED, and positive emotional intensity, *V* = 0.275, *F*(8, 418) = 8.327, *p* < 0.001, η*_*p*_*^2^ = 0.137. We conducted separate univariate ANOVAs on the outcome variables, which revealed significant effects on NED, negative emotional intensity, PED, and positive emotional intensity. See Table 2 for means, SDs, and difference tests. For NED, *post hoc* tests using *Hochberg’s GT2* showed that the two depressed groups had significantly lower NED than the healthy control group, *p*s < 0.05, but they did not differ from each other, *p* = 0.60. For negative emotional intensity, the three groups significantly varied from each other: The current depressed group had the highest levels, followed by the remitted group, with the healthy control group having the lowest levels, *p*s < 0.05. For PED, a pattern similar to NED emerged: The two depressed groups had significantly lower levels than the healthy control group, *p*s < 0.05, but the two depressed groups did not differ from each other, *p* = 0.95. In terms of positive emotional intensity, the current depressed group had significantly lower levels than the other two groups, *p*s < 0.01, who did not vary from each other, *p* = 0.85. Lastly, an analysis of covariance (ANCOVA), including verbal ability as a covariate, showed that the group effect was significant for NED, *F*(2, 206) = 4.596, *p* = 0.011, η*_*p*_*^2^ = 0.043, and PED, *F*(2, 207) = 4.496, *p* = 0.012, η*_*p*_*^2^ = 0.042.

**TABLE 2:** Emotion differentiation, emotional intensity, and open-ended responses of emotional experience by group.

	Healthy Control (*n* = 48)	Remitted Depressed (*n* = 80)	Current Depressed (*n* = 87)	

Experience Sampling Data				

	*M* (*SD*)	*M* (*SD*)	*M* (*SD*)	Difference Test (ANOVA)
NED	0.56 (0.28)_a_	0.46 (0.24)_b_	0.43 (0.29)_b_	*F*(2, 211) = 4.60, *p* = 0.010, η*_*p*_*^2^ = 0.042
Negative emotional intensity	0.34 (0.35)_a_	0.44 (0.29)_b_	0.73 (0.41)_c_	*F*(2, 212) = 20.61, *p* = 0.001, η*_*p*_*^2^ = 0.163
PED	−0.01 (0.33)_a_	−0.14 (0.24)_b_	−0.14 (0.35)_b_	*F*(2, 212) = 4.71, *p* = 0.010, η*_*p*_*^2^ = 0.043
Positive emotional intensity	1.61 (0.60)_a_	1.60 (0.56)_a_	1.16 (0.62)_b_	*F*(2, 212) = 10.68, *p* < 0.001, η*_*p*_*^2^ = 0.092

**Open-Ended Responses**				

	**Percentage^*d*^**	**Percentage**	**Percentage**	**Difference Test (Fisher’s Exact Test)**

**Emotion Terms**				
Negative	26.8_a_	39.7_a_	66.0_b_	Cramér’s *V* = 0.30, *p* < 0.001, 95% CI [0.20, 0.43]
Positive	59.8_a_	33.8_b_	14.9_c_	Cramér’s *V* = 0.36, *p* < 0.001, 95% CI [0.24, 0.47]
Neutral^e^	1.2	0	0	−
Overall (across valence)	73.2_a_	66.7_a_	76.6_a_	Cramér’s *V* = 0.09, *p* = 0.46, 95% CI [0.01, 0.24]
**General State Terms**				
Negative^e^	0	1.3	2.1	−
Positive	7.3_a_	9.0_a_	2.2_a_	Cramér’s *V* = 0.10, *p* = 0.34, 95% CI [0.04, 0.23]
Neutral	6.1_a_	14.1_a_	8.5_a_	Cramér’s *V* = 0.12, *p* = 0.23, 95% CI [0.03, 0.28]
Overall (across valence)	13.4_a_	21.8_a_	12.8_a_	Cramér’s *V* = 0.11, *p* = 0.30, 95% CI [0.01, 0.25]

*NED = negative emotion differentiation, PED = positive emotion differentiation.*

*Means and percentages with different subscripts within a row indicate significant pairwise comparison, *p* < 0.05.*

*^*d*^These are percentages of participants who responded to the open-ended question with a particular type of general state or emotion term in each diagnostic group; for example, 26.8% of the participants in the healthy control group responded with negative emotion term(s). ^*e*^We did not examine group differences in the use of neutral emotion terms or negative general state terms due to their low frequencies.*

### Open-Ended Emotional Responses

#### Likelihood of Term Use Across the Full Sample

To inform tests of Aim 2, we examined participants’ open-ended responses that described their momentary emotional experiences. Given that participants’ use of general state and emotion terms are paired categorical data, we conducted an exact McNemar’s test to examine participants’ relative use of emotion versus general state terms. Results suggested that, across valence, participants on average were significantly more likely to use emotion terms than general state terms to describe their momentary emotional experiences, *p* < 0.001. This pattern was true for experiences of negative valence and positive valence, *p*s < 0.001. However, participants were more likely to use general state terms than emotion terms to describe neutral experience, *p* < 0.001. Additionally, although participants infrequently used general state terms to describe their momentary emotional experiences overall, they were significantly more likely to use positive than negative general state terms, *p* < 0.001, but they were equally likely to use negative and positive emotion terms, *p* = 0.93.

#### Empirically Examining Theoretical Conceptualizations of Differentiation (Aim 2)

##### Association between differentiation and term use

To examine the association between differentiation and use of general state and emotion terms, we computed eight point-biserial correlations between NED and use of general state terms or emotion terms (i.e., negative, positive, neutral, and overall general state terms, as well as negative, positive, neutral, and overall emotion terms); we computed eight correlations for PED in a similar way. All correlation coefficients were small in magnitude and non-significant, ranging from −0.11 to 0.09 for NED and −0.13 to 0.04 for PED. This pattern of findings suggests a lack of correspondence between differentiation and use of general state or emotion terms.

##### Group differences in general state and emotion term use

We used Fisher’s exact tests to assess group differences in the likelihood of using general state and emotion terms overall (i.e., collapsing terms across positive, negative and neutral valence). This test examines whether the groups differ in the percentage of participants who used, for example, at least one general state term. See Table 2 for percentages and difference tests. The groups did not significantly differ in overall use of general state terms, *p* = 0.30. Similarly, we found no significant group effect on overall use of emotion terms, *p* = 0.46. We examined group differences in term use across valence because the groups significantly varied in negative and positive emotional intensity. That is, we did not examine whether groups differed in their use of general state terms and emotion terms by valence as this could reflect their different levels of emotional intensity. However, we still describe results of group differences in term use of a specific valence below, which are also summarized in Table 2.

Regarding general state terms, very few participants used negative general state terms. Consequently, we examined whether groups differed in the likelihood using positive and neutral general state terms. Results indicate that the three groups did not significantly differ in the likelihood of using positive or neutral general state terms.

Regarding group differences in use of emotion terms, because only one participant used a neutral emotion term, we only assessed group differences in the use of negative and positive emotion terms. There was a significant group difference in the likelihood of using negative emotion terms, *p* < 0.001. The current depressed group was more likely to use negative emotion terms than the remitted depressed group and healthy control group, who did not differ from each other (*p* = 0.095). For positive emotion terms, the three groups significantly differed from each other, *p* < 0.001. The current depressed group was the least likely to use positive emotion terms, followed by the remitted group, with the healthy control group most likely to use positive emotion terms. Given that Fisher’s exact test does not permit including covariates, we did not examine whether these group differences would hold after accounting for verbal ability.

## Discussion

A rich history documents the ways in which emotional functioning of people with current MDD varies from that of healthy controls (e.g., Houben et al., 2015), and many successful MDD treatments target these emotional disturbances (e.g., Greenberg and Watson, 2006). Despite advances in psychological and psychopharmacological treatments, the prevalence of MDD has not decreased in the last two decades (e.g., Jorm et al., 2017). One effective way to reduce the individual and societal burden of MDD is by decreasing its recurrence rates. Elucidating emotional disturbances that characterize those whose MDD is in remission may identify viable targets for primary and secondary prevention efforts. We focused on differentiation as one such target by investigating it in individuals whose MDD was in full remission, comparing them to a group with current depression and a healthy control group.

In terms of negative emotion, we found that compared to the healthy control group, the *current depressed group* had higher negative emotional intensity and lower NED. The negative emotional intensity findings are consistent with the diagnostic criteria of MDD (American Psychiatric Association, 2013) and many other studies (e.g., Watson et al., 1988; also see Thompson et al., 2021a). The current depressed group having lower NED than the healthy control group replicates Demiralp et al. (2012) and may help clarify associations between NED and depression, which have not been entirely consistent. NED may only be associated with depressive psychopathology when examining a wide range of current depressive symptoms, such as in the present study (also see Demiralp et al., 2012).

This is the first investigation to examine NED in a sample whose MDD was in full remission. We found that the levels of NED in the *remitted depressed group* are diminished compared to the healthy control group, which is consistent with our hypothesis. In addition, the two depressed groups had similarly diminished NED, providing evidence that low NED is not a state effect of being in a depressive episode. Diminished NED could represent a more chronic feature of MDD, which may be a risk factor for MDD that exists outside depressive episodes or something that emerges during an episode and lasts even after the episode remits (i.e., a scar; Burcasa and Iacono, 2007). Prospective longitudinal research could track people who are at elevated risk for depression to see if NED predicts the onset of MDD. Preliminary evidence on depressive symptoms suggests that this might be the case (Rieffe and De Rooij, 2012; Liu et al., 2020), and if so, NED could represent a risk or vulnerability factor for the onset of MDD and be a viable target for prevention efforts. Another interpretation of these findings is that NED is only diminished in samples whose current depressive symptoms are above a certain threshold. Based on the current findings, that threshold may be the depressive symptom level that divides the healthy control and remitted depressed groups, the latter of which showed elevated current depressive symptoms than healthy controls. In contrast, at lower levels of severity, there may not be a straightforward association between NED and depressive symptoms. Accumulating research points to an interaction between NED and various risk factors in predicting a host of negative psychological outcomes (Seah and Coifman, 2021). For example, diminished NED predicted increases in depressive symptoms only in combination with high levels of brooding (Starr et al., 2017).

In terms of positive emotion, the current depressed group had significantly lower positive emotional intensity than the remitted and control groups, who did not vary from each other. These findings are consistent with the diagnostic criteria of MDD (American Psychiatric Association, 2013) and long history of research on positive emotional intensity and MDD status (e.g., Watson et al., 1988). PED was lower in the current depressed group than in the healthy control group, which is inconsistent with research findings indicating that depressive symptoms are unrelated to PED (e.g., Starr et al., 2017) and that those with current MDD do not vary in PED from healthy controls (Demiralp et al., 2012). Notably, NED and PED were positively correlated in the current study, but they were uncorrelated in Demiralp et al. (2012). This may be attributed to different positive emotions being assessed in these two studies. The present study included six positive emotions that represent a variety of arousal levels; in contrast, Demiralp et al. (2012) sampled four positive emotions that represent moderate to high levels of arousal (i.e., happy, excited, alert, and active). Future research should investigate if differentiation among high arousal positive emotions is not diminished in those with MDD. Another explanation could involve the age of the samples. The present sample included adults who were between 18 and 77 years old with an average age of 44.3 years, which is older than the sample in Demiralp et al. (2012) that averaged 27.8 years and did not include participants over 40 years old. Relatedly, research has found that age is positively associated with NED (Mankus et al., 2016), as well as many other putatively adaptive dimensions of emotion (e.g., emotional stability; Carstensen et al., 2011). Of course, although these ideas are speculative, they highlight the importance of research continuing to elucidate PED in clinical samples, consistent with recommendations by Thompson et al. (2021b).

We also examined emotional intensity in the remitted depressed group, and the findings largely serve as a replication of the extant literature. The remitted depressed group experienced levels of negative emotional intensity that were lower than the current depressed group but higher than the healthy control group. This pattern of findings is consistent with research comparing those with remitted MDD versus healthy controls (e.g., Wichers et al., 2012) as well as research comparing those with remitted versus current depressive disorders (Schoevers et al., 2020). In terms of positive emotional intensity, the remitted depressed group did not differ from the healthy control group, consistent with the majority of the extant literature (e.g., Wichers et al., 2012). Also replicating past work (Schoevers et al., 2020), those with remitted MDD had higher positive emotional intensity than those with current MDD. Because the intensity findings followed an expected pattern, they suggest that our sample is comparable to other samples, lending more confidence in the novel findings from this study.

In addition to conducting diagnostic interviews to assess depressive disorders, we assessed depressive symptoms using two measures–the MASQ anhedonic depression scale and the CES-D. Consistent with prior evidence (e.g., Starr et al., 2017), PED was not associated with depressive symptoms (as assessed by either measure). Interestingly, lower NED was significantly associated with higher CES-D, but it was not significantly associated with MASQ anhedonic depression, indicating that the link between NED and depressive symptoms may vary based on the depression measure. The current findings could help elucidate the role of depressive symptom measures in explaining the mixed findings on the association between NED and depressive symptoms.

One possible explanation for the discrepant findings across the two depressive symptoms measures is that they tap different aspects of depressive psychopathology (Nitschke et al., 2001; Bredemeier et al., 2010). Whereas the MASQ anhedonic depression scale focuses on symptoms unique to depression (i.e., anhedonia and low positive affect), the CES-D covers a wider range of symptomatology, including those that are non-specific to depression and anxiety, such as high negative emotional intensity (Clark and Watson, 1991; Buckby et al., 2007). It may be that the depressive symptomatology captured by the CES-D but not MASQ anhedonic depression, such as high negative emotional intensity, is driving its associations with NED. In fact, NED was no longer associated with CES-D when negative emotional intensity was taken into account, which is in line with Dejonckheere et al. (2019) argument that differentiation lacks explanatory power in predicting psychological well-being beyond negative emotional intensity (i.e., negative affect). As such, inconsistency in controlling for emotional intensity in past research, along with other reasons such as the range of depressive symptoms in the sample and the choice of depression measures, could explain some of the mixed findings. It is important to note, however, that NED has been significantly associated with depressive symptoms even after accounting for negative emotional intensity (e.g., Starr et al., 2017). These complex patterns speak of the need for future research to further clarify how NED is associated with depression, anxiety, and their overlapping features.

Our exploratory aim was to test predominant theoretical conceptualization of differentiation–whether individuals with lower differentiation use more general state terms and fewer emotion terms. Specifically, we examined (a) the correspondence between differentiation and participants’ use of general state and emotion terms, and (b) whether the depressed groups, who have lower differentiation relative to the healthy control group, would be more likely to use general state and less likely to use emotion terms. Our experience sampling protocol, like almost all others, used a list of emotion terms to assess momentary experience (Thompson et al., 2021b). The list did not include general state terms, the use of which would characterize low differentiation according to multiple conceptualizations (Kashdan et al., 2015). Therefore, we had participants generate their own descriptions of a momentary experience using an open-response format, which we coded for the presence of general state and emotion terms.

Regarding the correlations between differentiation and term use, neither NED nor PED were significantly associated with the use of any general state or emotion terms. Moreover, because both depressed groups had diminished NED and PED, according to predominant differentiation theory, they should show higher overall use of general state terms and lower overall use of emotion terms when describing their momentary emotional experience compared to a healthy control group. Inconsistent with this prediction, no group differences emerged for the overall use of general state and emotion terms (across valence). Therefore, these patterns of findings reflect a lack of correspondence between differentiation (as assessed via repeated measurements and computed using ICCs) and how the predominant theorizing of differentiation describes the use of general state and emotion terms.

This lack of correspondence between theoretical conceptualization and measurement of differentiation may be partly due to our assessment of general state and emotion terms at one point of time, which assessed momentary emotional experience. In contrast, computing ICCs across a series of momentary emotion ratings more likely reflects trait or global differentiation. Others have examined momentary and trait differentiation and found that they do not always align in their associations with well-being measures, suggesting the importance of considering the time frame within which differentiation is measured (Tomko et al., 2015; Erbas et al., 2021). However, it is also likely that the theoretical conceptualization of differentiation and its common measure tap distinct constructs. In fact, Ottenstein and Lischetzke (2020) measured trait differentiation by computing ICC as well as by aggregating a series of open-responses (i.e., proportion of specific affective state out of specific plus general affective states) and found that these two measures were unrelated (Study 2). Similarly, Williams and Uliaszek (2021) also measured NED via ICC and coding of open-ended descriptions of emotional experience, finding that these two measures were not significantly related. Thus, more research, especially using repeated open-ended measures to assess the use of general state and emotions terms, is needed in this area.

We also examined group differences in the use of general state and emotion terms by valence. Regarding general state terms, no group differences emerged for the use of positive or neutral general state terms (Group differences in negative general state terms were not examined due to their low frequency). Regarding emotion terms, the current depressed group was more likely to use negative emotion terms than the other two groups. The healthy control group was most likely to use positive emotion terms, followed by the remitted depressed group, with the current depressed group being the least likely to use positive emotion terms (Group differences in neutral emotion terms were not examined due to their low frequency). Readers should keep in mind that the open-ended responses assessed momentary emotion, not differentiation *per se*, and that there were some group differences in negative and positive emotional intensity. Consequently, interpreting these findings in the context of differentiation theory is complicated. For example, the current depressed group used the fewest positive emotion terms, but we cannot tease apart whether this is driven by the current depressed group’s diminished *positive* emotional intensity or low tendency to use positive *emotion* terms. To compare group difference patterns in differentiation and term use within a particular valence (e.g., between PED and positive emotion terms), future researchers could explicitly ask participants to report on their positive and negative emotion or restrict participants’ free response to a valence of interest.

The current research also informs how verbal ability is implicated in differentiation. Replicating Ottenstein and Lischetzke (2020), verbal ability was not significantly associated with NED in the present study. Verbal ability was also not significantly associated with PED either, extending this literature to examine PED. Although Ottenstein and Lischetzke did not find significant relations between verbal ability and their open-ended assessment of differentiation (i.e., specificity index), we found significant associations between verbal ability and the use of certain categories of general state and emotion terms. We find these significant findings particularly surprising because our open-ended measure of momentary emotion was not designed to assess differentiation *per se*, and we coded responses in a straightforward way–whether participants’ descriptions contained general state terms and emotion terms. That is, our coding scheme did not take into account the specificity, nuance, or complexity of terms. For example, the emotion terms sad and bittersweet were coded similarly. We also did not compute any sort of ratio of these two categories; that is, scores for general state and emotion terms were considered independently. Despite this, findings suggest that verbal ability is more strongly implicated when participants provide open-ended responses (versus making Likert type ratings of emotions). It will be useful for future research to further explore the relation between verbal ability and differentiation given the proliferation of studies examining differentiation using open-ended responses (e.g., Williams and Uliaszek, 2021) and implication for the conceptualization of differentiation (Thompson et al., 2021b).

Though the present study was novel in many ways and extends the literature on differentiation and depression, we want to note a few additional limitations. First, given that the present study consisted of one wave of data collection, we cannot rule out that NED and PED were diminished before the onset of MDD. Consequently, the temporal nature of the association between NED and MDD is unclear and requires further investigation. Second, although the study’s hypotheses are couched in theory and existing research, we did not pre-register their hypotheses. Third, the open-ended format we used to assess participants’ momentary emotional experience was administered as part of an online survey participants completed outside the laboratory. Because participants may have completed the survey when they are in certain emotional states (e.g., neutral, calm), this study design may have resulted in sampling a narrower range of emotional experiences than had we utilized repeated sampling (e.g., Ottenstein and Lischetzke, 2020) or a mood induction (Williams and Uliaszek, 2021). In addition, because this measure was only administered once, we could not assess certain psychometric properties (e.g., reliability).

In conclusion, the present study contributes to the literature on differentiation by including participants with remitted MDD. Further, by also including those with current depression, our sample represented a wide range of depressive psychopathology assessed via diagnostic interviewing, addressing limitations in many differentiation and depression studies (i.e., assessing depression using self-report measures, using relatively healthy samples; Matt et al., 2016). Finding that both current and remitted depressed groups have diminished NED and PED suggests that low differentiation may be a vulnerability factor for MDD or a lasting consequence of the disorder itself (i.e., a scar). Future research using longitudinal designs to elucidate the temporal associations between diminished differentiation with the onset and recurrence of MDD will inform whether interventions targeting differentiation may be useful in reducing the risk for the onset or recurrence of MDD. Finally, our exploratory data provides further evidence (see Ottenstein and Lischetzke, 2020) of a lack of correspondence between the predominant theoretical conceptualization and its common measurement. Considering the mounting evidence that differentiation is linked to well-being, including depression, it is critical for future research to clarify the concept of differentiation and its appropriate measurements.

## Data Availability Statement

The datasets presented in this study can be found in online repositories. The names of the repository/repositories and accession number(s) can be found below: https://osf.io/6hb9f/?view_only=efec095bf84c44dfa1b30403fe0b8bb0.

## Ethics Statement

The studies involving human participants were reviewed and approved by the Institutional Review Board at Washington University in St. Louis. The patients/participants provided their written informed consent to participate in this study.

## Author Contributions

RT and MB developed the study concept. RT supervised the performance of data collection. RT, DL, and MB performed the data analysis. RT, DL, and ES drafted the manuscript. MB provided critical revisions. All authors contributed to the study design and approved the final version of the manuscript for submission.

## Conflict of Interest

The authors declare that the research was conducted in the absence of any commercial or financial relationships that could be construed as a potential conflict of interest.

## Publisher’s Note

All claims expressed in this article are solely those of the authors and do not necessarily represent those of their affiliated organizations, or those of the publisher, the editors and the reviewers. Any product that may be evaluated in this article, or claim that may be made by its manufacturer, is not guaranteed or endorsed by the publisher.

## References

[B1] American Psychiatric Association (2013). *Diagnostic and statistical manual of mental disorders*, 5th Edn. Washington, DC: American Psychiatric Association.

[B2] Andrade L. Caraveo-Anduaga J. J. Berglund P. Bijl R. V. Graaf R. D. Vollebergh W. (2003). The epidemiology of major depressive episodes: results from the International Consortium of Psychiatric Epidemiology (ICPE) Surveys. *Int. J. Methods Psychiatric Res.* 12 3–21. 10.1002/mpr.138 12830306 PMC6878531

[B3] Augustine A. A. Larsen R. J. (2012). Is a trait really the mean of states? Similarities and differences between traditional and aggregate assessments of personality. *J. Indiv. Diff.* 33 131–137. 10.1027/1614-0001/a000083

[B4] Barrett L. F. Gross J. Christensen T. C. Benvenuto M. (2001). Knowing what you’re feeling and knowing what to do about it: Mapping the relation between emotion differentiation and emotion regulation. *Cogn. Emot.* 15 713–724. 10.1080/02699930143000239

[B5] Barrett L. F. Mesquita B. Ochsner K. N. Gross J. J. (2007). The experience of emotion. *Annu. Rev. Psychol.* 58 373–403. 10.1146/annurev.psych.58.110405.085709 17002554 PMC1934613

[B6] Barrett L. F. Russell J. A. (1999). The structure of current affect: Controversies and emerging consensus. *Curr. Direc. Psychol. Sci.* 8 10–14. 10.1111/1467-8721.00003

[B7] Bockting C. L. Hollon S. D. Jarrett R. B. Kuyken W. Dobson K. (2015). A lifetime approach to major depressive disorder: The contributions of psychological interventions in preventing relapse and recurrence. *Clin. Psychol. Rev.* 41 16–26. 10.1016/j.cpr.2015.02.003 25754289

[B8] Boden M. T. Thompson R. J. Dizén M. Berenbaum H. Baker J. P. (2013). Are emotional clarity and emotion differentiation related? *Cogn. Emot.* 27 961–978. 10.1080/02699931.2012.751899 23237455

[B9] Bredemeier K. Spielberg J. M. Silton R. L. Berenbaum H. Heller W. Miller G. A. (2010). Screening for depressive disorders using the mood and anxiety symptoms questionnaire anhedonic depression scale: A receiver-operating characteristic analysis. *Psychol. Assess.* 22 702–710. 10.1037/a0019915 20822283 PMC2992834

[B10] Buckby J. A. Yung A. R. Cosgrave E. M. Killackey E. J. (2007). Clinical utility of the Mood and Anxiety Symptom Questionnaire (MASQ) in a sample of young help-seekers. *BMC Psychiatry* 7:50. 10.1186/1471-244X-7-50 17868477 PMC2151061

[B11] Burcasa S. L. Iacono W. G. (2007). Risk for recurrence in depression. *Clin. Psychol. Rev.* 27 959–985. 10.1016/j.cpr.2007.02.005 17448579 PMC2169519

[B12] Carstensen L. L. Turan B. Schiebe S. Ram N. Ersner-Hershfield H. Samanez-Larkin G. R. (2011). Emotional experience improves with age: Evidence based on over 10 years of experience sampling. *Psychol. Aging* 26 21–33. 10.1037/a0021285 20973600 PMC3332527

[B13] Clark L. A. Watson D. (1991). Tripartite model of anxiety and depression: Psychometric evidence and taxonomic implications. *J. Abnormal Psychol.* 100 316–336. 10.1037//0021-843x.100.3.316 1918611

[B14] Dejonckheere E. Mestdagh M. Houben M. Rutten I. Sels L. Kuppens P. (2019). Complex affect dynamics add limited information to the prediction of psychological well-being. *Nat. Hum. Behav.* 3 478–491. 30988484 10.1038/s41562-019-0555-0

[B15] Demiralp E. Thompson R. J. Mata J. Jaeggi S. Buschkuehl M. Barrett L. F. (2012). Feeling blue or turquoise? Emotional differentiation in major depressive disorder. *Psychol. Sci.* 23 1410–1416. 10.1177/0956797612444903 23070307 PMC4004625

[B16] Dixon-Gordon K. L. Chapman A. L. Weiss N. H. Rosenthal M. Z. (2014). A preliminary examination of the role of emotion differentiation in the relationship between borderline personality and urges for maladaptive behaviors. *J. Psychopathol. Behav. Assess.* 36 616–625. 10.1007/s10862-014-9423-4 25750478 PMC4346340

[B17] Eaton W. W. Alexandre P. Bienvenu O. J. Clarke D. Martins S. S. Zablotsky B. (2012). “The burden of mental disorders,” in *Public mental health*, ed. Eaton W. W. (Oxford: Oxford University Press), 1–14.

[B18] Eaton W. W. Smith C. Ybarra M. Muntaner C. Tien A. (2004). “Center for Epidemiologic Studies Depression Scale: Review and revision (CESD and CESD-R),” in *The use of psychological testing for treatment planning and outcomes assessment: Instruments for adults*, Vol. 3 ed. Maruish M. E. (Mahwah, NJ: Lawrence Erlbaum), 363–377.

[B19] Erbas Y. Ceulemans E. Pe M. L. Koval P. Kuppens P. (2014). Negative emotion differentiation: Its personality and well-being correlates and a comparison of different assessment methods. *Cogn. Emot.* 28 1196–1213. 10.1080/02699931.2013.875890 24410047

[B20] Erbas Y. Kalokerinos E. Kuppens P. van Halem S. Ceulemans E. (2021). Momentary emotion differentiation: The derivation and validation of a framework to study within-person fluctuations in emotion differentiation. *Assessment* 10.1177/1073191121990089 [Epub ahead of print]. 33522259

[B21] Faul F. Erdfelder E. Lang A.-G. Buchner A. (2007). G^∗^Power 3: A flexible statistical power analysis program for the social, behavioral, and biomedical sciences. *Behav. Res. Methods* 39 175–191. 10.3758/BF03193146 17695343

[B22] Figueroa C. A. Mocking R. J. Mahmoud G. A. Koeter M. W. Bockting C. L. van der Does W. (2018). The measurement of cognitive reactivity to sad mood in patients remitted from major depressive disorder. *Br. J. Clin. Psychol.* 57 313–327. 10.1111/bjc.12175 29488231 PMC6099317

[B23] First M. B. Williams J. B. W. Karg R. S. Spitzer R. L. (2015). *Structured clinical interview for DSM-5–Research Version (SCID-5-RV, Version 1.0.0).* Washington, DC: American Psychiatric Association.

[B24] Fredrickson B. L. (2001). The role of positive emotions in positive psychology: The broaden-and-build theory of positive emotions. *Am. Psychol.* 56 218–226. 10.1037/0003-066X.56.3.218 11315248 PMC3122271

[B25] Giraudeau B. (1996). Negative values of the intraclass correlation coefficient are not theoretically possible. *J. Clin. Epidemiol.* 49:1205. 10.1016/0895-4356(96)00053-4 8827004

[B26] Goldston R. B. Gara M. A. Woolfolk R. L. (1992). Emotion differentiation: A correlate of symptom severity in major depression. *J. Nervous Mental Dis.* 180 712–718. 10.1097/00005053-199211000-00005 1431822

[B27] Gotlib I. H. Joormann J. (2010). Cognition and depression: current status and future directions. *Annu. Rev. Clin. Psychol.* 6 285–312. 10.1146/annurev.clinpsy.121208.131305 20192795 PMC2845726

[B28] Greenberg L. S. Watson J. C. (2006). *Emotion-focused therapy for depression.* Washington, DC: American Psychological Association.

[B29] Gross J. J. Muñoz R. F. (1995). Emotion regulation and mental health. *Clin. Psychol. Sci. Practice* 2 151–164. 10.1111/j.1468-2850.1995.tb00036.x

[B30] Grühn D. Lumley M. A. Diehl M. Labouvie-Vief G. (2013). Time-based indicators of emotional complexity: Interrelations and correlates. *Emotion* 13 226–237. 10.1037/a0030363 23163712 PMC3600390

[B31] Houben M. Van Den Noortgate W. Kuppens P. (2015). The relation between short-term emotion dynamics and psychological well-being: A meta-analysis. *Psychol. Bull.* 141 901–930. 10.1037/a0038822 25822133

[B32] Jorm A. F. Patten S. B. Brugha T. S. Mojtabai R. (2017). Has increased provision of treatment reduced the prevalence of common mental disorders? Review of the evidence from four countries. *World Psychiatry* 15 90–99. 10.1002/wps.20388 28127925 PMC5269479

[B33] Kashdan T. B. Barrett L. F. McKnight P. E. (2015). Unpacking emotion differentiation: Transforming unpleasant experience by perceiving distinctions in negativity. *Curr. Direc. Psychol. Sci.* 24 10–16. 10.1177/0963721414550708

[B34] Kessler R. C. Berglund P. Demler O. Jin R. Koretz D. Merikangas K. R. (2003). The epidemiology of major depressive disorder: Results from the National Comorbidity Survey Replication (NCS-R). *J. Am. Med. Assoc.* 289 3095–3105. 10.1001/jama.289.23.3095 12813115

[B35] Lennarz H. K. Lichtwarck-Aschoff A. Timmerman M. E. Granic I. (2018). Emotion differentiation and its relation with emotional well-being in adolescents. *Cogn. Emot.* 32 651–657. 10.1080/02699931.2017.1338177 28602148

[B36] Liu D. Y. Gilbert K. E. Thompson R. J. (2020). Emotion differentiation moderates the effects of rumination on depression: A longitudinal study. *Emotion* 20 1234–1243. 10.1037/emo0000627 31246044 PMC6933110

[B37] Liu D. Y. Thompson R. J. (2017). Selection and implementation of emotion regulation strategies in major depressive disorder: An integrative review. *Clin. Psychol. Rev.* 57 183–194. 10.1016/j.cpr.2017.07.004 28739277

[B38] Mankus A. M. Boden M. T. Thompson R. J. (2016). Sources of variation in emotional awareness: Age, gender, and socioeconomic status. *Personal. Indiv. Diff.* 89 28–33. 10.1016/j.paid.2015.09.043 26500384 PMC4612349

[B39] Matt L. M. Fresco D. M. Coifman K. G. (2016). Trait anxiety and attenuated negative affect differentiation: A vulnerability factor to consider? *Anxiety Stress Coping* 29 685–698. 10.1080/10615806.2016.1163544 26961095

[B40] McHugh M. L. (2012). Interrater reliability: The kappa statistic. *Biochem. Med.* 23 276–282. 10.11613/BM.2012.031 PMC3900052 23092060

[B41] Morin A. J. Moullec G. Maiano C. Layet L. Just J. L. Ninot G. (2011). Psychometric properties of the Center for Epidemiologic Studies Depression Scale (CES-D) in French clinical and nonclinical adults. *Rev. Epidemiol. Sante. Publique* 59 327–340. 21925817 10.1016/j.respe.2011.03.061

[B42] Muhtadie L. Akinola M. Koslov K. Berry Mendes W. (2015). Vagal flexibility: A physiological predictor of social sensitivity. *J. Personal. Soc. Psychol.* 109 106–120. 10.1037/pspp0000016 25545841 PMC4478218

[B43] Nezlek J. B. (2017). A practical guide to understanding reliability in studies of within-person variability. *J. Res. Personal.* 69 149–155. 10.1016/j.jrp.2016.06.020

[B44] Nitschke J. B. Heller W. Imig J. C. McDonald R. P. Miller G. A. (2001). Distinguishing dimensions of anxiety and depression. *Cogn. Ther. Res.* 25 1–22. 10.1023/A:1026485530405

[B45] O’Toole M. S. Renna M. E. Elkjær E. Mikkelsen M. B. Mennin D. S. (2019). A systematic review and meta-analysis of the association between complexity of emotion experience and behavioral adaptation. *Emot. Rev.* 12 23–38. 10.1177/1754073919876019

[B46] Ottenstein C. (2020). Emotion regulation effectiveness accounts for the associations of self-reported emotion differentiation with well-being and depression. *Cogn. Emot.* 34 994–1002. 10.1080/02699931.2019.1691506 31726942

[B47] Ottenstein C. Lischetzke T. (2020). Development of a novel method of emotion differentiation that uses open-ended descriptions of momentary affective states. *Assessment* 27 1928–1945. 10.1177/1073191119839138 30947508 PMC7545652

[B48] Plonsker R. Gavish Biran D. Zvielli A. Bernstein A. (2017). Cognitive fusion and emotion differentiation: does getting entangled with our thoughts dysregulate the generation, experience and regulation of emotion? *Cogn. Emot.* 31 1286–1293. 10.1080/02699931.2016.1211993 27484697

[B49] Pond R. S. Jr. Kashdan T. B. DeWall C. N. Savostyanova A. Lambert N. M. Fincham F. D. (2012). Emotion differentiation moderates aggressive tendencies in angry people: A daily diary analysis. *Emotion* 104 326–337. 10.1037/a0025762 22023359

[B50] Radloff L. S. (1977). The CES-D Scale: A self-report depression scale for research in the general population. *Appl. Psychol. Measure.* 1 385–401. 10.1177/014662167700100306 26918431

[B51] Rieffe C. De Rooij M. (2012). The longitudinal relationship between emotion awareness and internalising symptoms during late childhood. *Eur. Child Adolesc. Psychiatry* 21 349–356. 10.1007/s00787-012-0267-8 22466448 PMC3370159

[B52] Schoevers R. A. van Borkulo C. D. Lamers F. Servaas M. N. Bastiaansen J. A. Beekman A. T. F. (2020). Affect fluctuations examined with ecological momentary assessment in patients with current or remitted depression and anxiety disorders. *Psychol. Med.* 51 1–10. 10.1017/S0033291720000689 32234092 PMC8381239

[B53] Schwarz N. (2012). “Why researchers should think “real-time”: A cognitive rationale,” in *Handbook of research methods for studying daily life*, eds Mehl M. R. Conner T. S. (New York: The Guilford Press), 22–42.

[B54] Seah T. H. Coifman K. G. (2021). Emotion differentiation and behavioral dysregulation in clinical and non-clinical samples: A meta-analysis. *Emotion* 2021:968. 10.1037/emo0000968 34264705

[B55] Shiota M. N. Neufeld S. L. Danvers A. F. Osborne E. A. Sng O. Yee C. I. (2014). Positive emotion differentiation: A functional approach. *Soc. Personal. Psychol. Compass* 8 104–117. 10.1111/spc3.12092

[B56] Shrout P. E. Fleiss J. L. (1979). Intraclass correlations: Uses in assessing rater reliability. *Psychol. Bull.* 86 420–428. 10.1037/0033-2909.86.2.420 18839484

[B57] Starr L. R. Hershenberg R. Li Y. I. Shaw Z. A. (2017). When feelings lack precision: Low positive and negative emotion differentiation and depressive symptoms in daily life. *Clin. Psychol. Sci.* 5 613–631. 10.1177/2167702617694657

[B58] Taylor P. J. (2010). *An Introduction to intraclass correlation that resolves some common confusions.* Boston, MA: University of Massachusetts.

[B59] Thompson R. J. Bailen N. English T. (2021a). Everyday emotional experiences in major depressive disorder in remission: An experience sampling study. *Clin. Psychol. Sci.* 10.1177/2167702621992082 [Epub ahead of print].

[B60] Thompson R. J. Springstein T. Boden M. T. (2021b). Gaining clarity about emotion differentiation. *Soc. Personal. Psychol. Compass* 15:e12584. 10.1111/spc3.12584

[B61] Tomko R. L. Lane S. P. Pronove L. M. Treloar H. R. Brown W. C. Solhan M. B. (2015). Undifferentiated negative affect and impulsivity in borderline personality and depressive disorders: A momentary perspective. *J. Abnormal Psychol.* 124 740–753. 10.1037/abn0000064 26147324 PMC4573801

[B62] Watson D. Clark L. A. Carey G. (1988). Positive and negative affectivity and their relation to anxiety and depressive disorders. *J. Abnormal Psychol.* 97 346–353. 10.1037/0021-843X.97.3.346 3192830

[B63] Watson D. Weber K. Assenheimer J. S. Clark L. A. Strauss M. E. McCormick R. A. (1995). Testing a tripartite model: I. Evaluating the convergent and discriminant validity of anxiety and depression symptoms scales. *J. Abnormal Psychol.* 104 3–14. 10.1037/0021-843X.104.1.3 7897050

[B64] Weinberger A. H. Gbedemah M. Martinez A. M. Galea S. Goodwin R. D. (2018). Trends in depression prevalence in the USA from 2005 to 2015: Widening disparities in vulnerable groups. *Psychol. Med.* 48 1309–1315. 10.1017/S0033291717002781 29021005

[B65] Weissman M. M. Sholomskas D. Pottenger M. Prusoff B. A. Locke B. Z. (1977). Assessing depressive symptoms in five psychiatric populations: A validation study. *Am. J. Epidemiol.* 106 203–214. 900119 10.1093/oxfordjournals.aje.a112455

[B66] Weschler D. (1997). *Weschler Adult Intelligence Scale*, 3rd Edn. San Antonio, TX: The Psychological Corporation.

[B67] Wichers M. Peeters F. Rutten B. P. F. Jacobs N. Derom C. Thiery E. (2012). A time-lagged momentary assessment study on daily life physical activity and affect. *Health Psychol.* 31 135–144. 10.1037/a0025688 21988094

[B68] Widdershoven R. L. Wichers M. Kuppens P. Hartmann J. A. Menne-Lothmann C. Simons C. J. P. (2019). Effect of self-monitoring through experience sampling on emotion differentiation in depression. *J. Affect. Disord.* 244 71–77. 10.1016/j.jad.2018.10.092 30321767

[B69] Williams G. E. Uliaszek A. A. (2021). Measuring negative emotion differentiation via coded descriptions of emotional experience. *Assessment* 69 149–155. 10.1177/10731911211003949 33794656

[B70] Willroth E. C. Flett J. A. M. Mauss I. B. (2020). Depressive symptoms and deficits in stress-reactive negative, positive, and within-emotion-category differentiation: A daily diary study. *J. Personal.* 88 174–184. 10.1111/jopy.12475 30927441 PMC6768767

